# Performance and Scalability of Discriminative Metrics for Comparative Gene Identification in 12 *Drosophila* Genomes

**DOI:** 10.1371/journal.pcbi.1000067

**Published:** 2008-04-18

**Authors:** Michael F. Lin, Ameya N. Deoras, Matthew D. Rasmussen, Manolis Kellis

**Affiliations:** 1 Broad Institute of MIT and Harvard University, Cambridge, Massachusetts, United States of America; 2 Computer Science and Artificial Intelligence Laboratory, Massachusetts Institute of Technology, Cambridge, Massachusetts, United States of America; Centre de Regulació Genòmica (CRG), Spain

## Abstract

Comparative genomics of multiple related species is a powerful methodology for the discovery of functional genomic elements, and its power should increase with the number of species compared. Here, we use 12 *Drosophila* genomes to study the power of comparative genomics metrics to distinguish between protein-coding and non-coding regions. First, we study the relative power of different comparative metrics and their relationship to single-species metrics. We find that even relatively simple multi-species metrics robustly outperform advanced single-species metrics, especially for shorter exons (≤240 nt), which are common in animal genomes. Moreover, the two capture largely independent features of protein-coding genes, with different sensitivity/specificity trade-offs, such that their combinations lead to even greater discriminatory power. In addition, we study how discovery power scales with the number and phylogenetic distance of the genomes compared. We find that species at a broad range of distances are comparably effective informants for pairwise comparative gene identification, but that these are surpassed by multi-species comparisons at similar evolutionary divergence. In particular, while pairwise discovery power plateaued at larger distances and never outperformed the most advanced single-species metrics, multi-species comparisons continued to benefit even from the most distant species with no apparent saturation. Last, we find that genes in functional categories typically considered fast-evolving can nonetheless be recovered at very high rates using comparative methods. Our results have implications for comparative genomics analyses in any species, including the human.

## Author Summary

Comparing the genomes of related species is a powerful approach to the discovery of functional elements such as protein-coding genes. Theoretically, using more species should lead to more discovery power. Many questions remain, however, surrounding the optimal choice of species to compare and how to best use multi-species alignments. It is even possible that practical limitations in the sequencing, assembly, and alignment of genomes could effectively negate the benefit of using more species. Here, we used 12 complete fly genomes to study a variety of metrics used to identify protein-coding genes, including methods that analyze only the genome of interest and comparative methods that examine evolutionary signatures in genome alignments. We found that species over a surprisingly broad range of phylogenetic distances were effective in comparative analyses, and that discovery power continued to scale with each additional species without apparent saturation. We also examined whether comparative methods systematically miss genes considered fast-evolving, and studied how performance is influenced by genome alignment strategies. Our results can help guide species selection for future comparative studies and provide methodological guidance for a variety of gene identification tasks, including the design of future *de novo* gene predictors and the search for unusual gene structures.

## Introduction

The recent availability of complete genome sequences from many closely related species has enabled the use of comparative genomics for systematic gene identification. In practice, the discovery power of comparative genomics is intrinsically linked to specific methods for extracting information from from multi-species alignments. Numerous such methods have been developed for gene identification, capturing diverse signals that distinguish protein-coding genes from non-coding regions. These signals are found in the primary sequence of the target genome (e.g. nucleotide frequencies and codon usage biases) and also in the distinctive evolutionary signatures of protein-coding regions (e.g. favoring synonymous vs. non-synonymous substitutions) that only become apparent when informant species are used for comparison.

In this paper, we study the discovery power of diverse discriminative metrics that capture comparative genomics as well as single-species evidence. Given a region of the genome and, when available, its alignment across multiple species, discriminative metrics produce a score that indicates how likely the region is to be protein-coding. Similar to previous studies of the performance of single-sequence metrics [1]–[3], we measure discovery power in a binary classification framework, based on each metric's ability to discriminate between known protein-coding exons and random non-coding regions.

The goals of our study are twofold. First, we seek to determine the relative power of different metrics, their independence, and the power obtained by combining them. Such metrics can be applied to assess and correct existing gene annotations [4],[5], and to decide whether experimentally derived cDNA sequences represent protein-coding mRNAs or non-coding transcripts [6],[7]. In addition, our study is immediately applicable to the design of discriminative features for comparative gene structure predictors that can incorporate artibrary metrics to determine precise exon boundaries, such as systems based on semi-Markov conditional random fields (SMCRFs). While initial studies on such discriminative gene prediction systems have successfully focused on their training algorithms [8]–[10] and advantages over their generative predecessors [11],[12], here we focus on the discriminative features they can use, which ultimately enable their increased power.

Second, we seek to understand how discovery power scales with the phylogenetic distance and number of species compared. On one hand, increasing either distance or number of species should, in principle, provide more signal and therefore increased discovery power [13], as shown in several pilot studies in selected genomic regions [14]–[18]. On the other hand, greater phylogenetic distance and more informant species can also lead to conflicting evidence arising from elements that have undergone evolutionary divergence. Moreover, additional species may in practice result in increased noise and systematic errors in the sequencing, assembly, and alignment of complete genomes. In fact, initial studies using *de novo* gene structure predictors with multiple informants led to mixed results [19],[20]. Thus, empirical studies of the scalability of gene identification power in multiple complete genomes are needed, to help address several remaining questions surrounding comparative gene identification that are still unresolved: is there an optimal pairwise distance for gene identification, does multi-species discovery power saturate after a small number of compared species, are some classes of genes systematically missed by comparative methods, are synteny-anchored alignments necessary for achieving high specificity?

To address these two goals, we have assembled a large benchmark dataset consisting of tens of thousands of coding and non-coding sequences aligned across twelve recently sequenced *Drosophila* genomes [21],[22]. We measure the discriminatory power of diverse metrics and how it varies with sequence length, phylogenetic distance, total number of informant sequences, and the genome alignment strategy. We also study the redundancy and independence of different metrics, and the discovery power of metric combinations. Finally, we discuss the overall strategic implications of our results for comparative approaches to gene identification.

### Discriminative Metrics for Gene Identification

We evaluate both well-known methods for gene identification as well as several metrics that we have developed. These metrics are briefly summarized here and in Table 1, while we provide full implementation details in the Methods section.

**Table 1:** Discriminative metrics for gene identification.

	Metric	Description	References
Pairwise comparative	*K_A_*/*K_S_*	Ratio of non-synonymous to synonymous substitutions per site	[30],[31]
	Codon Substitution Frequencies (CSF)	Log-likelihood ratio of coding vs. non-coding based on empirical frequencies of all codon substitutions	[5]
	Reading Frame Conservation (RFC)	Percent of nucleotides in same reading frame offset based on indel pattern	[4],[32]
	TBLASTX	Significance of protein sequence similarity (bit score), independent of genome alignments	[33]
	Seq. conservation	(baseline) Percent identity	-
Multi-species comparative	*dN/dS* test	Pr(*dN/dS* < 1), probability that synonymous substitution rate exceeds non-synonymous substitution rate, based on maximum likelihood phylogenetic models	[34],[35],[36]
	Codon Substitution Frequencies (CSF)	Pairwise CSF log-likelihood ratios combined by median in each column	[5]
	Reading Frame Conservation (RFC)	Pairwise RFC scores for each informant combined by voting scheme	[4],[32]
	Seq. conservation	(baseline) Averaged identity in each column	-
Single sequence	Fourier transform	Three-base periodicity in genetic code	[37]
	Codon bias	Unequal usage of synonymous codons	[38]
	Interpolated context models (ICMs)	Generative probabilistic models measuring *k*-mer frequency biases	[39]
	Z curve	Linear discriminant analysis on *k*-mer frequencies	[2]

Additional details are provided in Methods.

### Pairwise comparative metrics

Most initial efforts at comparative gene identification used a single informant genome to support the annotation of a target genome [15], [23]–[29]. We selected several metrics that capture the essential properties of coding sequence evolution that they observe: the *K_A_*/*K_S_* ratio [30],[31] and the Codon Substitution Frequencies (CSF) score [5] observe biases towards synonymous and other conservative codon substitutions; the Reading Frame Conservation (RFC) score observes the strong bias of indels within coding regions to be multiples of three in length [4],[32]; TBLASTX measures the genome-wide significance of protein sequence similarity [33]; finally, a baseline sequence conservation metric simply measures the percent nucleotide identity between the target and informant sequences.

### Multi-species comparative metrics

We also selected several metrics that use multi-species alignments: the *dN*/*dS* test observes biases towards synonymous codon substitution using a statistical test based on maximum likelihood phylogenetic algorithms [34]–[36]; the multi-species CSF and RFC scores use *ad hoc* strategies to efficiently combine their respective pairwise scores; lastly, a baseline multi-species sequence conservation metric measures the largest fraction of species having the same nucleotide in each column (plurality), averaged across the alignment.

### Single-sequence metrics

We also included several single-sequence metrics in our benchmarks to compare them to the comparative methods. Since previous studies have benchmarked many single-sequence metrics extensively [1]–[3], we chose only a representative set here: the Fourier transform measures the strength of the three-base periodicity in coding sequences [37]; codon bias observes the unequal usage of synonymous codons, resulting in part from how different synonymous codons affect translation efficiency [38]; interpolated context models (ICMs) are generative probabilistic models that observe reading frame-dependent biases in the frequencies of *k*-mers in coding sequences, simultaneously for several different *k*-mer sizes [39]; lastly, Z curve observes reading frame-dependent biases in *k*-mer frequencies using a discriminative approach based on Fisher linear discriminant analysis [2].

### Benchmarks for Gene Identification Metrics in 12 Fly Genomes

To benchmark the discriminatory power of each of these metrics, we assembled a test set consisting of 10,722 known protein-coding exons (from 2,734 genes) in the fruit fly *Drosophila melanogaster*, and 39,181 random intergenic regions with the same length and strand distribution (see Methods). These provide an ideal setting in which to evaluate genome-wide comparative genomics methods given the high quality of the FlyBase gene annotations [5] and the recent sequencing of ten *Drosophila* genomes [21],[22], in addition to *D. melanogaster* [40] and *D. pseudoobscura* [41]. We extracted each of these regions from two different sets of whole-genome sequence alignments of the twelve fly genomes [22], one generated by MULTIZ [42], which uses local alignments of high-similarity regions, and the second generated by the Mercator orthology mapper (C. Dewey and L. Pachter) and MAVID sequence aligner [43], based on the identification of orthologous segments in each genome by conserved gene order (synteny).

For each metric, we scored all the 49,903 regions in our test set (10,722 exons and 39,181 non-coding regions) and then measured its ability to correctly classify them as coding or non-coding. We used four-fold cross-validation to train and apply the metrics that require training data. We evaluated the performance of each metric by examining receiver-operator characteristic (ROC) curves showing its sensitivity and specificity at different score cutoffs. (Here and throughout this paper, we use the term *specificity* as it is defined in binary classification problems: the fraction of true negatives that are correctly classified as negative. This differs from the common usage of the term in the gene prediction field to refer to the fraction of the examples classified as positive that are true positives. Additionally, we use the term *false positive rate* to mean 1-Specificity, or the fraction of true negatives incorrectly classified as positive.)

Based on the ROC curve for each metric, we also computed two different summary error measures, to facilitate comparing the performance of different metrics and methodological choices:

- The *minimum average error* (MAE) is the average of the false negative rate and the false positive rate at the cutoff where this average is minimized; intuitively, this is the “elbow” of the ROC curve. This represents the fraction of examples that are incorrectly classified (if the positive and negative classes are the same size), at a single point on the ROC curve.
- The *area above the curve* (AAC) is the area lying above the ROC curve in the unit square. Although it lacks a simple interpretation, the AAC summarizes more information about classification performance over all sensitivity/specificity regimes, providing a measure complementary to MAE.

## Results

### Performance, Independence, and Combinations of the Metrics

**Figure 1:**
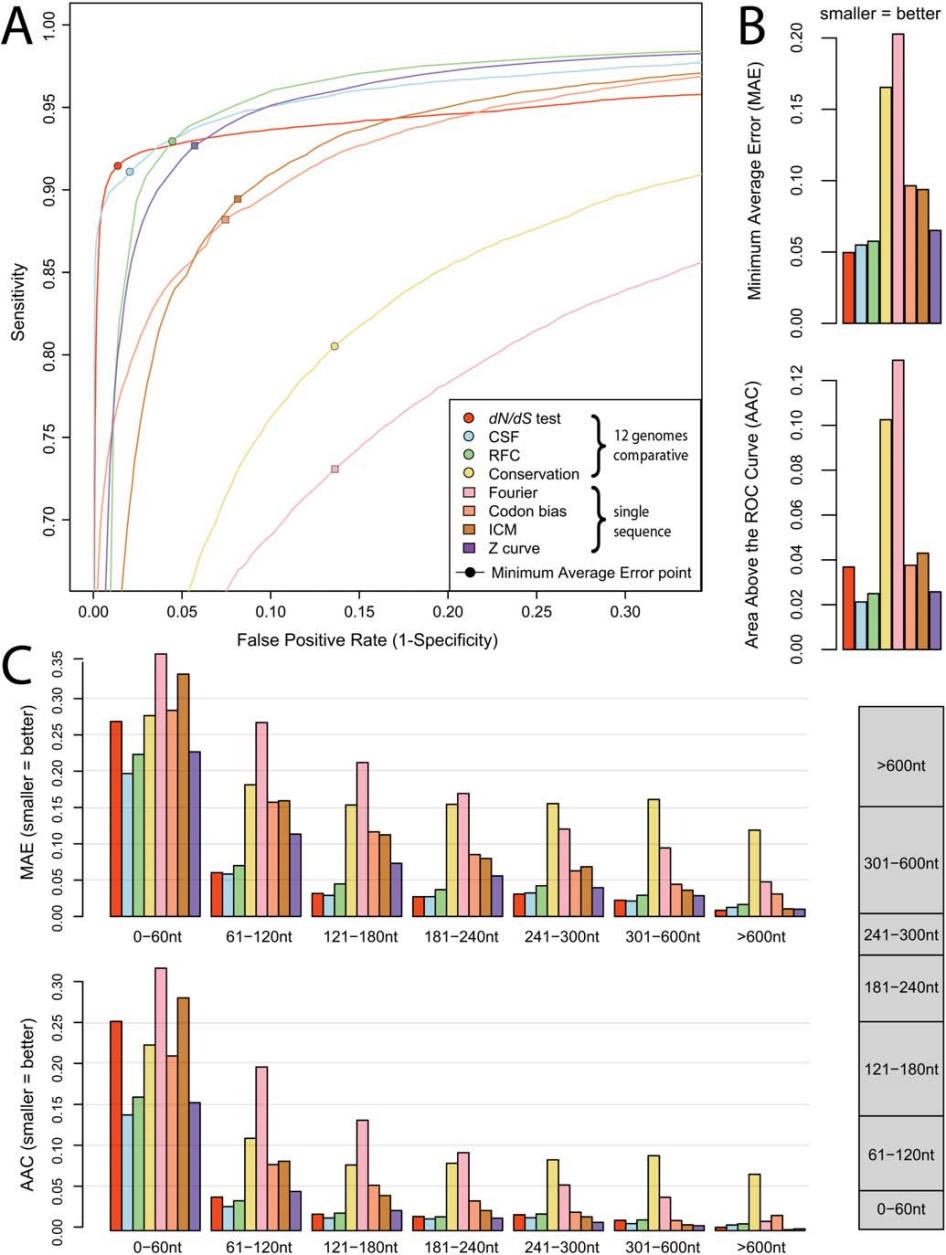
Overall discovery power of discriminative metrics using 12 genomes. (A) ROC curves showing sensitivity and specificity of each metric on classifying 10,722 known exons and 39,181 random non-coding regions. Comparative methods tended to outperform single-sequence metrics, with the exception of a baseline sequence conservation metric. CSF and the *dN*/*dS* test achieved near-perfect specificity, while RFC achieved high sensitivity. (B) Summary error statistics for each metric computed from the ROC curves. Minimum Average Error (MAE) is the minimum average of the false negative rate and false positive rate. Area Above the Curve (AAC) is the area above the ROC curve in the unit square. (C) MAE and AAC error statistics for each metric when the dataset is partitioned into several sequence length categories. All metrics tended to perform better on longer sequences than on shorter sequences. Comparative methods strongly outperformed single-sequence metrics on short sequences (60–240 nt). Inset: relative size of each sequence length category.

We first compared the overall performance of the metrics (Figure 1). All of the metrics we evaluated demonstrated high classification performance, but some general trends were apparent. The comparative metrics (using the MULTIZ alignments of all twelve fly genomes) generally outperformed the single-sequence metrics (except for the baseline sequence conservation metric). For example, the best comparative metric resulted in 24% lower error than the best single-sequence metric (0.050 MAE for the *dN*/*dS* test vs. 0.065 for Z curve). Different metrics were preferable at different sensitivity/specificity tradeoffs. For example, the CSF and *dN*/*dS* metrics achieved the highest specificity (99.9% for CSF) even at fairly high sensitivities (85.2%). RFC tended towards higher sensitivity and lower specificity than CSF and *dN*/*dS*.

We also compared the pairwise metrics, using the best pairwise informant (*D. ananassae*; we investigate different pairwise informants below), and found similar trends (Figure S1). For example, CSF and *K_A_*/*K_S_* performed comparably, showing the highest specificity, while RFC tended towards higher sensitivity and lower specificity. TBLASTX performed substantially worse than *K_A_*/*K_S_*, CSF, and RFC, but it was still better than our baseline conservation metric. Notably, none of the pairwise comparative metrics outperformed the best single-sequence metric (Z curve) according to MAE and AAC error, and they exhibited generally lower sensitivity. CSF and *K_A_*/*K_S_* were, however, able to achieve higher specificity at a moderate sensitivity tradeoff. For example, at 80% sensitivity, CSF had a nearly ten-fold lower false positive rate than Z curve (0.15% and 1.39%); the specificity of CSF exceeded Z curve at less than 85% sensitivity, compared to 93% sensitivity at Z curve's MAE point.

### Comparative methods are strongly preferred for short exons

We next assessed each metric's discriminatory power for different sequence length categories (Figure 1C). All of the metrics performed better on longer sequences than shorter sequences. Single-sequence metrics performed comparably or slightly better than comparative methods for long sequences (>240 nt), but comparative methods strongly outperformed single-sequence metrics on shorter sequences. For example, in the length range of 181–240 nt (which includes the median exon length) the best comparative metric resulted in 51% lower error than the best single-sequence metric (0.027 MAE for the *dN*/*dS* test and 0.056 MAE for Z curve). In the shorter length range of 121–180 nt, the best comparative metric resulted in 60% lower error than the best single-sequence metric (0.029 MAE for CSF and 0.073 MAE for Z curve). Different comparative methods were also preferred at different lengths. For example, CSF strongly outperformed the *dN*/*dS* test on the shortest sequences (≤60 nt), while they performed comparably on longer sequences.

### Independence of the metrics

**Figure 2:**
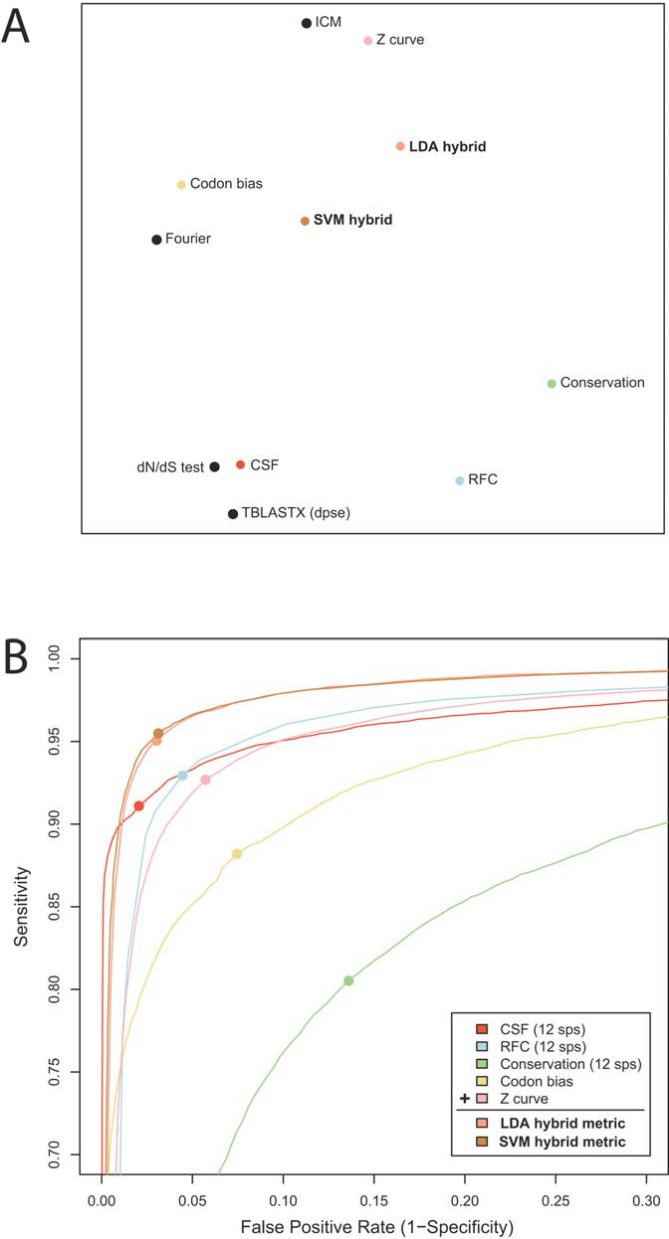
Independence of metrics and discovery power of metric combinations. (A) Multidimensional scaling (MDS) visualization in which each point represents a metric and the distance between any two points approximately represents their dissimilarity, measured as 1-(rank correlation of the scores of the known exons). Hybrid metrics appear closer to the center, suggesting that they successfully combine distinct information from the individual metrics. (B) ROC curves showing the performance of two hybrid metrics created by combining five comparative and single-sequence metrics using Linear Discriminant Analysis (LDA) or a Support Vector Machine (SVM). The hybrid metrics outperformed all of their input metrics.

While each of the metrics we studied exhibited unique performance characteristics, some measure similar fundamental lines of evidence, and thus may tend to err on the same examples. We investigated the independence of the metrics, indicated by how differently they rank the exons in our test set, using a dimensionality reduction technique called multidimensional scaling (MDS; see Methods). This analysis led to a two-dimensional visualization shown in Figure 2A, in which each point represents one of the metrics and the distance between the points approximately represents their dissimilarity.

We found that the *dN*/*dS* test and CSF behaved very similarly, while RFC was clearly distinct. The sequence conservation metric was separate from each of these, while TBLASTX clustered with CSF and *dN*/*dS*. The four single-sequence metrics formed two additional clusters distinct from the comparative metrics. These findings agree with intuition: CSF and the *dN*/*dS* test both observe the distinctive biases in codon substitutions in protein-coding sequences, while RFC observes patterns of insertions and deletions that are essentially orthogonal to codon substitutions, and the single-sequence metrics observe compositional biases and periodicities that are ignored by the comparative metrics.

### Combining metrics

The relative independence of several of the metrics suggests that combining them could lead to higher performance. We selected five metrics representing each of the MDS clusters (CSF, RFC, sequence conservation, Z curve, and codon bias) and combined them using cross-validated linear discriminant analysis (LDA). As expected, the hybrid metric outperformed any of its inputs: by MAE error, the LDA hybrid resulted in 27% lower error than its best input metric (0.040 MAE for LDA vs. 0.055 for CSF). The hybrid metric demonstrated much higher sensitivity than any of its input metrics (Figure 2B), and higher specificity than all of the input metrics except CSF. We obtained almost identical results using a second hybrid metric based on a linear support vector machine instead of LDA. Thus, although CSF and the *dN*/*dS* test remain the methods of choice for the highest specificity, the hybrid metrics achieved higher overall performance.

### Dependence of Comparative Methods on Genome Alignments

We next investigated how strongly the performance of the comparative methods depends on genome sequence alignments. We compared the above results, based on MULTIZ local similarity-based alignments, with the corresponding results based on the synteny-anchored Mercator/MAVID alignments. Overall, the two alignments led to highly concordant results, with similar trends in the performance of the metrics relative to each other and across different sequence lengths. There were, however, some notable differences in their absolute levels of performance.

We expected the local alignment approach to give higher sensitivity than the synteny-anchored alignments, since it should be better able to align exons that have undergone rearrangements [45]. Indeed, we found that MULTIZ tended to align more species for each region (Figure S2) and led to higher sensitivity than the Mercator/MAVID alignments (e.g. 90% vs. 87% for CSF at 99% specificity, with 85% of exons detected in both alignments; Figure S3). Conversely, we expected the synteny-anchoring approach used by Mercator/MAVID to give higher specificity than the local alignment approach of MULTIZ, since it may generate fewer spurious non-orthologous alignments [45]. However, we found that while the Mercator/MAVID alignment could lead to slightly higher specificity, it did so only at disproportionate sensitivity tradeoffs. For example, with the baseline sequence conservation metric, specificity using the Mercator/MAVID alignments exceeded that of the MULTIZ alignments only at lower than 58% sensitivity (compared to 80% sensitivity at the MULTIZ-based MAE point). Similarly, with RFC, specificity resulting from the Mercator/MAVID alignments was greater only at lower than 63% sensitivity (compared to 92% MAE sensitivity).

Overall, the Mercator/MAVID alignments led to somewhat lower sensitivity without a clear specificity advantage, and this was reflected in worse MAE and AAC error statistics (Figure S3). We therefore focused on the MULTIZ alignments for the remainder of our analysis. We note, however, that the Mercator/MAVID alignments did allow detection of some exons not detected in the MULTIZ alignments (∼2% of all exons). More generally, these empirical observations could be highly dependent on parameter settings of the genome alignment programs, and further investigation of these strategies is required.

### A Wide Range of Phylogenetic Distances Is Effective in Pairwise Analysis

**Figure 3:**
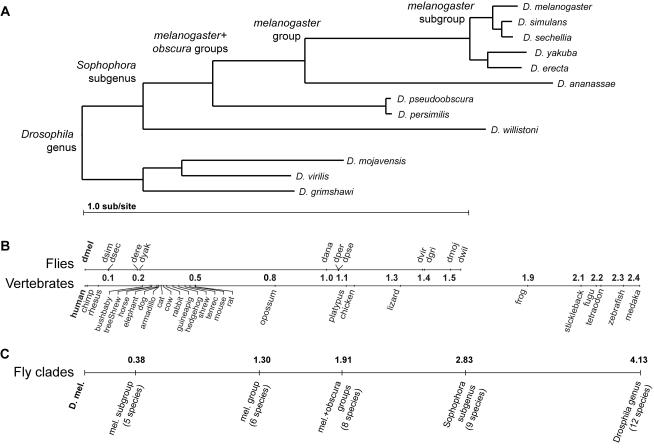
Evolutionary distances relating 12 *Drosophila* species. (A) Phylogenetic tree and estimated neutral branch lengths for the species. Tree topology follows the accepted phylogeny of these species [21],[22]. Neutral substitution rates estimated from 12,861 4-fold degenerate sites in syntenic one-to-one orthologs (see Methods). (B) Pairwise distance of each of the 11 other *Drosophila* species from *D. melanogaster*, as compared to similarly estimated distances for vertebrates. (C) Total independent branch length provided by several subsets of the *Drosophila* species used to benchmark multi-species methods.

To investigate which species are the most and least effective informants for gene identification, we evaluated each pairwise comparative metric using informant genomes at increasing evolutionary distance from *D. melanogaster*. We applied each metric to pairwise alignments of *D. melanogaster* with *D. erecta*, *D. ananassae*, *D. pseudoobscura*, *D. willistoni*, and *D. grimshawi*, each representing various clades within the genus *Drosophila* (Figure 3).

**Figure 4:**
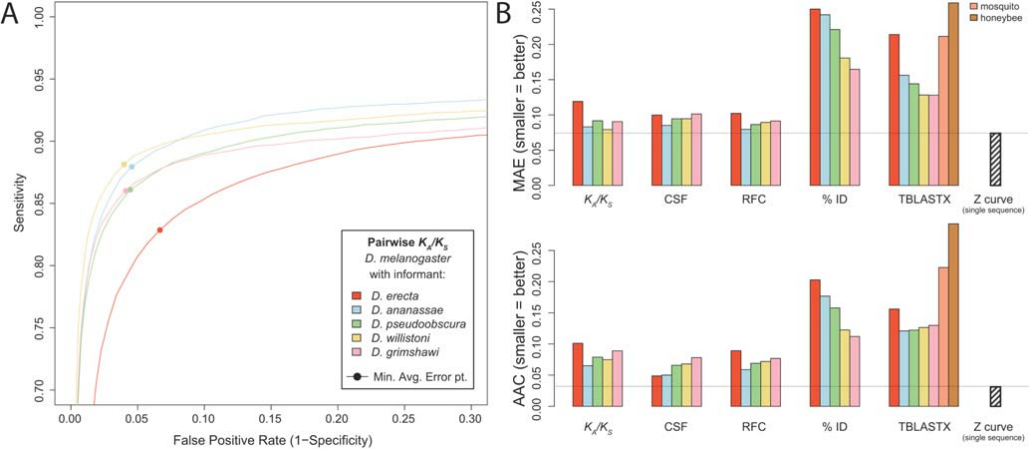
Pairwise discovery power using different informant species. (A) ROC curves for *K_A_*/*K_S_* using *D. melanogaster* with each of five different informant species. Species at a wide range of evolutionary distances performed comparably, except for *D. erecta*, the most closely related to *D. melanogaster*, which clearly underperformed the others. (B) MAE and AAC error statistics for each pairwise comparative metrics applied to the same five informants. *D. ananassae* (blue) is overall the preferred informant, but not uniformly so. For TBLASTX, the performance is also shown using mosquito (*Anopheles gambiae*) and honeybee (*Apis mellifera*), which led to worse performance than the *Drosophila* species. No pairwise comparison outperformed the best single-sequence metric (Z curve).

We found that *D. ananassae* was overall the most effective informant, outperforming other species on most metrics. However, inspection of the corresponding ROC curves often revealed a more complex situation, with multiple species showing similar performance, and sometimes higher for certain sensitivity/specificity tradeoffs. For example, with *K_A_*/*K_S_*, *D. ananassae* and *D. willistoni* performed comparably, with *D. ananassae* leading to slightly higher sensitivity and *D. willistoni* leading to slightly higher specificity (Figure 4A). Similarly, with RFC, closely related species led to slightly higher sensitivities, and more distant species led to slightly higher specificities (Figure S4). Hence, while *D. ananassae* was overall the most effective informant, it did not *robustly* outperform the other pairwise informants we studied. The only exception was *D. erecta*, the most closely related to *D. melanogaster* of the species we studied. *D. erecta* was consistently less informative than the others, leading to the lowest overall classification performance on most of the pairwise metrics.

To investigate more distant species for which we lacked whole-genome alignments, we also applied TBLASTX to the genomes of the mosquito [46] and honeybee [47]. We found that these species led to much worse performance than the *Drosophila* species as informants for *D. melanogaster* (Figure 4B).

We conclude that a broad range of species within the genus *Drosophila* (outside of the *melanogaster* subgroup) make effective pairwise informants for gene identification in *D. melanogaster*, while the mosquito and honeybee, the next most closely related species with fully sequenced genomes, are likely to be too distant for this application. These findings are consistent with a previous smaller-scale study of comparative gene identification power in flies [14], and previous theoretical and simulation studies suggesting that, while some mathematically optimal distance may exist, species at a broad range of phylogenetic distances should be comparably effective informants for identifying exons and other conserved elements [13],[15].

### Multi-Species Comparisons Lead to Higher Performance

We next investigated the effectiveness of increasing numbers of informant species on the metrics that can use multiple informants. We evaluated each metric using subsets of the available species corresponding to increasingly broad clades within the genus *Drosophila* (see phylogeny in Figure 3): the *melanogaster* subgroup (5 species including *D. melanogaster*), the *melanogaster* group (6 species), the *melanogaster* and *obscura* groups (8 species), the subgenus *Sophophora* (9 species), and finally all 12 species of the genus *Drosophila*.

**Figure 5:**
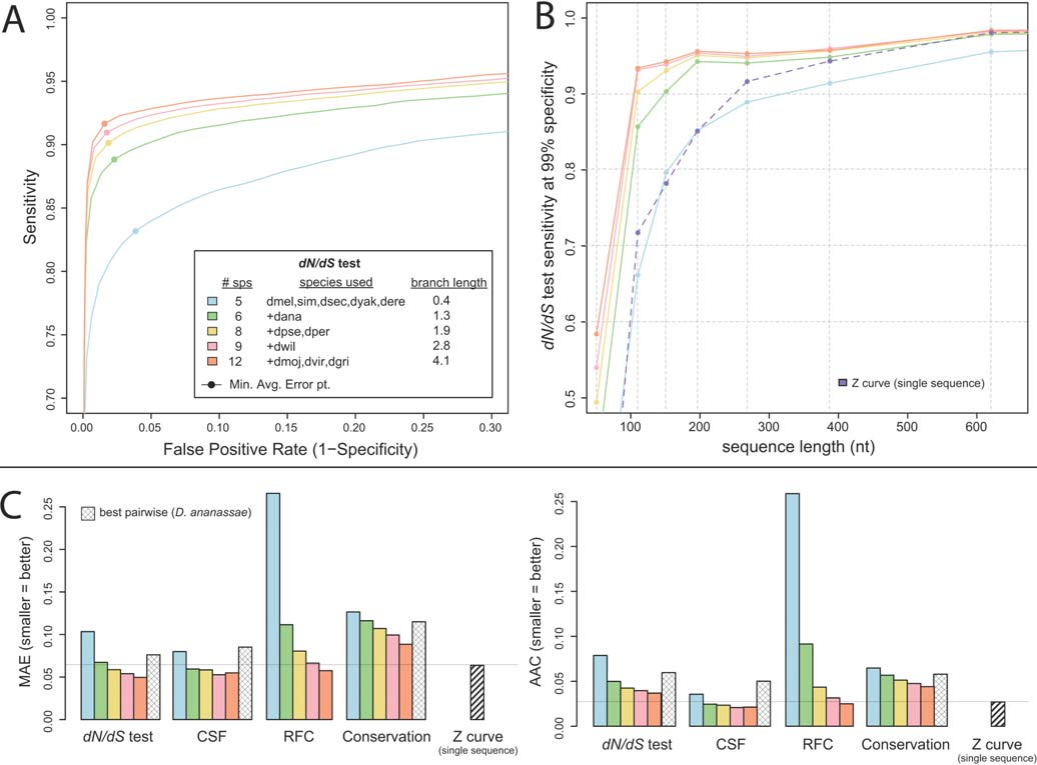
Multi-species discovery power using increasing numbers of informant species. (A) ROC curves for the *dN/dS* test using subsets of *Drosophila* species corresponding to increasingly broad phylogenetic clades from *D. melanogaster* (see Figure 1). Discriminatory power steadily increased as more informants were used, leading to strictly better sensitivity and specificity. (B) Effect of additional species was most pronounced for short exon lengths. (*x*-axis) mean length within a quantile of the sequence length distribution (*y*-axis) sensitivity of the *dN*/*dS* test within each quantile at fixed specificity (99%). (C) MAE and AAC error statistics for each multi-species comparative metric using the same subsets of informants. Also shown for comparison are the best pairwise analysis and the best single-sequence metric, both of which are outperformed by multi-species methods with sufficient informants.

We found that for each of the metrics we benchmarked in this way, discriminatory power tended to increase as additional informant species were used (Figure 5A). In contrast to our previous pairwise analysis, in which the most distant *Drosophila* informants led to similar or slightly worse performance than closer species, *adding* informants at increasing distances led to a clear trend in higher classification performance. The *dN*/*dS* test, RFC, and the sequence conservation metric each showed a smooth progression of increasing performance with each successively larger group of informant species. For example, starting from the four informants within the *melanogaster* subgroup, the *dN*/*dS* test achieved an MAE of 0.103. With the addition of each successive group of informants, the MAE was reduced relatively by 35%, 43%, 48%, and finally by 52%. CSF showed a similar trend through the subgenus *Sophophora*, but did not clearly benefit from the subsequent addition of the final three informants of subgenus *Drosophila*. In all cases, the improvement with multiple species was most pronounced for short exons (Figure 5B).

With a sufficient number of informants, the multi-species metrics surpassed single-sequence metrics according to MAE (Figure 5C). This also stands in contrast to our pairwise analysis, in which no informant enabled any comparative metric to outperform the best single-sequence metric (Z curve). CSF exceeded the performance of Z curve once we used at least six species (≥1.3 sub/site), *dN*/*dS* with at least eight species (≥1.9 sub/site), and RFC, using its simplistic vote-tallying scheme, with all twelve species (4.1 sub/site). The baseline sequence conservation metric never outperformed Z curve, although its performance also increased with additional species. (We note that while these results show that a certain number of informants is *sufficient*, they do not imply that they are all *necessary* to achieve some level of performance; removing informants that contribute very little independent branch length might not substantially reduce performance.)

In most cases, the four informants of the *melanogaster* subgroup together yielded worse performance than pairwise analysis with the best pairwise informant, *D. ananassae*. In contrast, all of the informant clades that combined *D. ananassae* with more distant species led to better performance than any pairwise analysis. This affirms our earlier conclusion, based on a pairwise analysis with *D. erecta*, that the species within the *melanogaster* subgroup are sub-optimal informants for the metrics we studied, presumably because they are too closely related to *D. melanogaster*. Indeed, the neutral distance of *D. ananassae* from *D. melanogaster* is 1.0 substitutions per neutral site, while the *total* independent branch length provided by the four *melanogaster* subgroup informants is only 0.4 sub/site.

### Characterizing Genes that Comparative Methods Fail to Detect

It is well-known that genes in certain categories of biological function tend to be faster-evolving [41], [46]–[48]. We lastly investigated whether comparative metrics therefore systematically fail to distinguish such genes from non-coding regions. We obtained Gene Ontology (GO) annotations [49],[50] for each of the 2,734 genes comprising our test set. For each of the 192 GO terms represented by at least thirty genes in our test set, we determined the fraction of those genes with at least one exon scoring above a stringent cutoff (“detected genes”).

We found that all of the functional categories we investigated had very high detection rates (Table S1). For example, with a CSF cutoff corresponding to 85% exon sensitivity and 99.9% specificity using all twelve fly genomes, the overall fraction of detected genes was 92%, and the detection rates surpassed 90% for all but two functional categories: serine-type endopeptidase activity (89% detected genes) and its superset, serine-type peptidase activity (86%). Serine proteases play key roles in insect innate immunity, and some likely evolve under positive selection [46],[51],[52]. Several other categories that intuition suggests might relate to more rapidly evolving genes, however, were not problematic, including immune response (94%), gametogenesis (95%) and G-protein coupled receptor activity (100%).

Instead, comparative metrics had the most difficulty detecting genes of unknown function. Three GO terms indicating unknown function (unknown cellular component, molecular function, and biological process) had only 67%, 61%, and 60% detected genes. In fact, of the genes that were not detected at this cutoff, 85% were of unknown function or lacked any GO term, compared to 49% of all the genes in our dataset. These trends held for all of the comparative metrics and cutoffs we investigated (Table S1).

Overall, these results indicate that comparative methods using the twelve fly genomes were able to detect the vast majority of genes in all of the functional categories we investigated (which were represented by at least 30 genes in our dataset; a larger sample might reveal more specific functional categories that are, in fact, very difficult for comparative methods to detect). They had much greater difficulty detecting genes of unknown function, which may be under less selective constraint overall [14],[21] but could also include a higher proportion of incorrect or spurious annotations [5]. Interestingly, Z curve, a single-sequence metric, also showed much lower sensitivity to genes of unknown function (Table S1), suggesting that these genes, if they are correctly annotated, tend to be unusual in several ways.

## Discussion

In this paper, we investigated discriminative metrics for distinguishing protein-coding sequences from non-coding sequences. We found that multi-species comparative methods outperform single-sequence metrics, particularly on short sequences (≤240 nt). On the other hand, the pairwise comparative methods we studied achieved higher specificity, but did not outperform advanced single-sequence metrics overall. We showed that several comparative and single-sequence metrics can be combined into a more powerful hybrid metric. We found that a broad range of species within the genus *Drosophila* are comparably effective pairwise informants for *D. melanogaster*, in agreement with theoretical predictions. We showed that adding more species to comparative analysis progressively increased genome-wide discovery power, for a variety of different methods. Contrary to expectation, we found no evidence that synteny-anchored alignments lead to appreciably higher specificity, and no evidence that comparative methods systematically fail to detect genes in functional categories typically considered fast-evolving.

Among the three multi-species comparative metrics we studied (CSF, the *dN*/*dS* test, and RFC; excluding the baseline sequence conservation metric), none strictly outperformed the others. RFC tended towards lower specificity but higher sensitivity than CSF and the *dN*/*dS* test. CSF was more effective than the *dN*/*dS* test on the shortest exons, but they performed comparably overall, and both achieved near-perfect specificity at moderate sensitivity tradeoffs. We developed CSF as a simpler alternative to the computationally expensive phylogenetic algorithms upon which the *dN*/*dS* test is based, and we consider it successful in this respect, considering its comparable results and its much faster total compute time (on our dataset, completed in several minutes for CSF vs. a few weeks for the *dN*/*dS* test using PAML).

On the other hand, our tests with different numbers of informant species suggest that the CSF method may benefit from future improvements to take advantage of ever-larger numbers of informants. Both CSF and RFC are discriminative methods that use heuristic approaches to combine multi-species evidence, making them less theoretically appealing than generative phylogenetic models such as those used in the *dN*/*dS* test. It is likely that such principled statistical frameworks can lead to further improvements for both CSF and RFC. Presently, however, the fact that both of these relatively simple methods outperformed advanced single-sequence metrics, and even competed with a maximum-likelihood phylogenetic algorithm, speaks to the power of the underlying comparative data. Lastly, we note that simple methods such as RFC and *K_A_*/*K_S_* might be preferable in certain ways when working with species for which high-accuracy training data is not available. In our setting, the best performing metrics tended to be highly parameterized approaches that require reliable training data, and thus probably benefited from the excellent FlyBase/BDGP annotation of the *D. melanogaster* genome.

### Selection of Informants for Comparative Gene Identification

Using a variety of different methods, we found that species ranging from 1.0–1.4 substitutions per neutral site from *D. melanogaster* are comparably effective informants for pairwise gene identification, with slight preference given to the closer end of this range. This “optimal” range might extend both towards closer species (between *D. erecta* and *D. ananassae*) and towards more distant species (between *D. grimshawi* and *A. gambiae*), but these distances were not explored in the currently sequenced genomes. This range is comparable to the distance from human of the opossum (0.8 sub/site), chicken (1.1 sub/site), and lizard (1.3 sub/site), suggesting that species more distant than the eutherian mammals (the farthest of which are less than 0.5 sub/site; Figure 3) may prove to be excellent informants for human gene identification.

Moreover, our study showed that comparative genomics power did not saturate with the number of species compared, as the multi-species metrics tended to show continued improvement from each progressively larger group of informants studied (Figure 5). The overall improvement did become more incremental as the number of informants grew, which could be interpreted either as diminishing returns from additional genomes, or simply as the expected asymptotic increase in performance towards an achievable optimum. Importantly, the improvement from more informants was far more pronounced among short exons than long exons (Figure 5B); this suggests that, while long exons are easy to discover even with few species, still more informants may significantly improve the discovery of short coding exons, and perhaps other classes of small elements. Thus, especially for small elements, we apparently have not yet reached a saturation point with twelve metazoan species spanning a total of 4.13 substitutions per neutral site.

We chose to express discovery power as a function of the neutral substitution rate estimated for the species compared (Figure 3). While this rate provides a compelling measure of expected discovery power [13], it is important to note that genetic distance between species (whether measured by neutral substitution rate or other metrics [21],[53]) is far from the only consideration that should guide comparative informant selection. For example, population dynamics affect the strength of selection relative to neutral drift, and thus may skew the relationship between neutral divergence and the significance of observed conservation in some lineages [54],[55]. Additionally, the genome size and the density and type of repetitive elements in an informant genome may affect the ability to sequence, assemble, and align it to a target genome, especially if low-coverage [18] or short-read [56],[57] sequencing strategies are used. Accurate alignment is further complicated by variation in the rates of chromosomal rearrangement and segmental duplication and loss, which are likely to affect the proportion of the genome that can be accurately recognized as orthologous, even for species that show similar nucleotide divergence.

Much more fundamentally, distant species share less in common biologically; indeed, the 12 *Drosophila* species were selected in part to represent the diverse ecological niches they occupy [58] and the neutral distance they span (approximately corresponding to the distance between human and reptiles). Thus, while our results suggest that such distant species may nonetheless be highly informative given high-quality sequences and alignments, future empirical studies should compare them to the use of many species at closer distances, such as those represented by the eutherian mammals, for gene identification.

### Implications for Gene Prediction Strategies

One application of the metrics we have studied will be their integration into *de novo* gene structure predictors based on semi-Markov conditional random fields, which can combine multiple discriminative metrics in a manner not unlike our LDA hybrid. Our results suggest that these systems should be able to use multiple informant species and multiple metrics to identify protein-coding sequences with higher accuracy, especially on short exons. Still, it is not obvious that these trends in the metrics' performance necessarily imply higher-accuracy prediction of complete gene structures, since the latter also strongly depends on the detection of splice sites and other sequence signals [12],[59]. Additionally, like the more advanced metrics we studied, such systems tend to be highly parameterized and thus dependent on high-quality training data, which may not be available in less well-studied species. More fundamentally, the probabilistic models used in gene predictors make simplifying assumptions about gene structures that lead to many incorrect predictions, and that cannot be relaxed just by using more powerful metrics. For example, they currently cannot predict nested and interleaved genes, which are fairly common in metazoan genomes [5], [50], [60]–[62], since these structures violate Markov independence assumptions. A similar challenge is presented by alternative splice isoforms with mutually exclusive exons that do not splice to each other in-frame.

The methods we have studied also have other important applications, such as assessing and refining existing annotations, and searching the genome for coding regions that are systematically missed or erroneously modeled by other methods. In particular, the effectiveness of comparative methods for detecting short coding regions may prove crucial in identifying short proteins, which are known to serve important biological roles but have probably been systematically under-represented in genome annotations [63]–[66]. They also provide a promising way to search for gene structures that violate traditional assumptions entirely, such as stop codon readthrough, translational frameshifts and polycistronic transcripts, which also might be more common in animal genomes than currently appreciated [5].

## Methods

### Genomes, Alignments, Annotations, and Phylogeny

We used “Comparative Analysis Freeze 1” assemblies of the twelve *Drosophila* genomes [21] available from the following web site: http://rana.lbl.gov/drosophila/assemblies.html. We used two different genome alignment sets [22]. One was derived from a synteny map generated by Mercator (C. Dewey, http://www.biostat.wisc.edu/~cdewey/mercator/) and sequence alignments generated by MAVID [43]. The other genome alignments were generated by MULTIZ [42]. These alignments are available from the following web site: http://rana.lbl.gov/drosophila/wiki/index.php/Alignment.

We obtained FlyBase release 4.3 annotations from the following web site:

ftp://ftp.flybase.net/genomes/Drosophila_melanogaster/dmel_r4.3_20060303/gff/.

We estimated branch lengths in the phylogenetic tree for the flies (shown in Figure 3) based on four-fold degenerate sites in alignments of orthologous protein-coding genes. We identified one-to-one orthologs based on FlyBase annotation release 4.3 for *D. melanogaster* and community annotations for the 11 other species [21], yielding 12,861 four-fold sites. Then, to estimate branch lengths, we ran PHYML v2.4.4 [67] with an HKY model of sequence evolution, a fixed tree topology (Figure 3A), and remaining parameters at default values. For comparison with vertebrates, we estimated the branch lengths for 28 vertebrates using 10,340 four-fold sites, based on alignments of genes with one-to-one orthologs in human, dog, and mouse [68]. We obtained the MULTIZ vertebrate alignments from the UCSC Genome Browser [69].

### Dataset Preparation

We randomly sampled 2,734 of the 13,733 euchromatic genes in FlyBase annotation release 4.3, and then selected all 10,722 non-overlapping exons of all transcripts of those genes. We chose this strategy of randomly sampling genes and selecting all exons of those genes, rather than directly sampling exons, to facilitate studying how the power of each metric varies across different functional categories of genes. Although not by design, the length distribution of sequences in our test set (median = 224 nt, mean = 404 nt, sd = 570 nt) is very similar to the length distribution of exons in the genome (median = 220 nt, mean = 408 nt, sd = 568 nt). Each known exon was evaluated in its annotated reading frame.

For each known exon in our dataset, we selected four non-coding regions of the same length and strand. We selected each of these regions by randomly choosing a start coordinate in the BDGP Release 4 assembly of the *D. melanogaster* euchromatic chromosome arms, and ensuring that the resulting region did not overlap an annotated coding exon. We also chose only regions consisting of at least 50% nucleotide characters (as opposed to Ns). The codon reading frame for the non-coding regions was always set arbitrarily to 0 (that is, they were always considered to begin with a complete codon). We removed in-frame stop codons in *D. melanogaster* from the non-coding regions (the length of each control region matched the corresponding exon *after* removing stop codons). All the regions in the dataset were selected without regard to how well they were aligned in either genome alignment set we used.

The coordinates, sequences, and alignments of our dataset are available for download (Text S1).

### Metric Training and Evaluation

CSF and the single-sequence metrics (except for Fourier transform) require training to estimate parameters. To avoid overfitting, we trained and applied them using four-fold cross validation: we randomly partitioned the dataset into four subsets, and then generated scores for each subset by training on the other three subsets. We then combined the scores for the subsets to obtain scores for the entire dataset. We applied the other metrics directly to each sequence.

We computed ROC curves for each metric by choosing 250 cutoffs representing quantiles of the score distribution over the entire dataset, and at each cutoff, evaluating sensitivity and specificity when sequences scoring above the cutoff are considered positively classified, and sequences scoring less than or equal to the cutoff are negatively classified. Some metrics failed to produce a score for some sequences; for example, comparative metrics produced no score for sequences in which no alignment was present. These sequences were regarded as negatively classified at all cutoffs, reflecting a non-coding default hypothesis. Our ROC curves may therefore underestimate the sensitivity or overestimate the specificity that each comparative method would exhibit if given perfect alignments of all orthologous elements.

We computed the MAE as the highest average sensitivity and specificity among the 250 points on the ROC curve, and the AAC by trapezoidal integration over these points.

### Metric Implementation Details

#### K_A_/K_S_

To estimate *K_A_*/*K_S_*, we used the method of Nei and Gojobori [30], which is simple and widely used although it is known to have certain inherent biases [31]. We considered only codons with ungapped alignments between *D. melanogaster* and the informant.

#### TBLASTX

We used the blastall program in NCBI BLAST 2.2.15 [33] with the parameters -p tblastx -m 9 against the repeat-masked genome assembly of the informant species. For each sequence, we used the best “bit score” among the resulting hits as the score for that sequence. We applied TBLASTX to the mosquito and honeybee in addition to the *Drosophila* species. We obtained these genome assemblies [46],[47] from the UCSC Genome Browser [69], assembly versions anoGam1 and apiMel2.

#### dN/dS test

We carried out the *dN*/*dS* test by using PAML 3.14 [34] to compute likelihoods of each sequence alignment under the assumption of either *dN*/*dS* = 1 or *dN*/*dS* estimated by maximum likelihood. Each multiple sequence alignment was pre-processed to make it acceptable to PAML as follows: gaps in the *D. melanogaster* sequence were removed, ends were trimmed so that the sequence only contains complete codons, and in-frame stop codons were changed to gaps in the informant sequences. Additionally, rows (informant species) with more than 50% gapped positions were removed, to reduce the computational cost of marginalizing over such heavily gapped rows.

PAML was then run twice on each alignment, once with fix_omega = 1 and once with fix_omega = 0. The other paramaters, common to both runs, were runmode = 0, seqtype = 1, CodonFreq = 2, model = 0. The tree was specified as shown in Figure 3. The log-likelihood values computed by the two runs were subtracted to obtain a log likelihood ratio used as the score for the region.

For practical reasons, PAML was not allowed to run for more than one hour on any individual alignment. Cases in which PAML exceeded this time limit, where no informant sequences remained after preprocessing, or otherwise failed were regarded as negatively classified at all cutoffs. This occurred in only 70 of 49,903 cases with 12 flies and 242 of 49,903 cases with the *melanogaster* subgroup informants.

#### CSF

The CSF metric is based on estimates of the frequencies at which all pairs of codons are substituted between genes in the target species and the informants [5]. First, let us consider computing the score for a pairwise alignment only. Consider the alignment of a putative ORF/exon as two sequences of codons *A* and *B*, where *A_k_* is the target codon that aligns to the informant codon *B_k_* at position *k* in the target codon sequence (position 3*k* in the in-frame target nucleotide sequence). CSF assigns a score to each codon position *k* where: (1) *A_k_* and *B_k_* are both un-gapped triplets, (2) *A_k_* is not a stop codon, and (3) *A_k_*≠*B_k_*. CSF then sums these scores to obtain an overall score for the sequence.

The score assigned to a codon substitution (*a*,*b*) is a log-likelihood ratio indicating how much more frequently that substitution occurs in coding regions than in non-coding regions. Each likelihood compared in this ratio is derived from a Codon Substitution Matrix (CSM), where
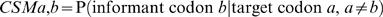



The entries of the CSM are estimated for each target and informant by counting aligned codon pairs in training data, and then normalizing the rows to obtain the desired conditional probabilities. We train two CSMs, one for which the training data is alignments of known protein-coding genes (*CSM^C^*) and one for which the training data is alignments of random non-coding regions (*CSM^N^*). The score that CSF assigns a codon substitution (*a*,*b*) is then
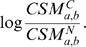



With multiple informants, CSF uses an *ad hoc* strategy to combine evidence from the informants without double-counting multiple apparent substitutions among extant species that result from fewer evolutionary events in their ancestors. For each target codon position *k*, CSF assigns a score to codon substitutions between the target and each informant exactly as in the pairwise case, using the appropriate CSMs for each informant. CSF then takes the median of these scores to obtain a composite score for position *k*, and sums these composite scores to obtain an overall score for the sequence. Note that the median is usually taken on fewer than *n* pairwise scores, since the pairwise scores are only assigned to ungapped informant codons that differ from the target codon.

#### RFC

We applied the RFC metric exactly as previously described [4],[32]. Briefly, given an alignment of a region of the target genome (*D. melanogaster*), a pairwise score between the target and each informant wass computed as the percentage of target nucleotides that aligned in the same reading frame in the informant (taking the largest such percentage out of the three possible reading frame offsets). With multiple informant species, each species votes +1, −1, or 0 based on a species-specific cutoff on the pairwise RFC score: +1 if the score is above, −1 if the score is below, or 0 if there was no sequence aligned. These votes are then summed to obtain an overall score for the region. The cutoff for each species is chosen by examining the typically bimodal distribution of the score between known coding and non-coding regions, and usually ranges between 70% and 80%.

#### Sequence conservation metrics

The pairwise sequence conservation metric is simply the percent identity between the target and informant sequences (as a fraction of the target sequence length). For multiple alignments, we assigned a score to each target nucleotide column corresponding to the largest fraction of species having the same nucleotide in that column (plurality), and averaged these scores across the columns of the alignment.

#### Fourier transform

The Fourier transform metric is an aggregate measure of the three-base periodicities of each nucleotide character in coding sequences [37],[70]. First, the DNA sequence is converted into four binary indicator sequences, one for each nucleotide, e.g.
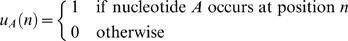



For each nucleotide, a three-base periodicity is then calculated by computing the magnitude of the discrete Fourier transform (DFT) of its indicator sequence at 1/3 frequency, e.g.
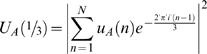



The overall score of the sequence is then computed by summing the contribution of each nucleotide periodicity normalized by the length of the sequence,
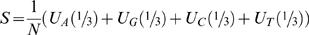



We found that the discriminative performance of this metric is identical to that obtained by computing the signal-to-noise ratio of the 1/3 frequency component of the DFT [71]. We chose the former because it has fewer free parameters.

#### Codon bias

Let *a_i_* be the amino acid translation of codon *i*. The metric utilizes codon usage vectors *C* and *N* for coding and non-coding sequences, where *C_i_* is the likelihood of codon *i* conditional on amino acid *a_i_* in coding regions, and *N_i_* is the corresponding likelihood for non-coding regions. *C_i_* is estimated from training data by determining the ratio of the number of times codon *i* occurs in-frame to the total number of times amino acid *a_i_* occurs in-frame; *N_i_* is estimated similarly with an arbitrary frame. To evaluate a given sequence, a total log-likelihood ratio *LLR* is computed by summing 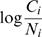 for each putative in-frame codon *i* in the sequence. *LLR* is positive if the codon bias in the given sequence is more similar to the coding regions in the training set than to the non-coding regions, and negative otherwise.

#### ICMs

We used Glimmer 3.02 [39] to build and evaluate the ICMs. In the training step, we used the build-icm program to estimate parameters for coding and non-coding ICMs. For both models, we used the default *depth* = 6. We found a choice of *width* = 6 improved discrimination over the default setting. The coding ICM was trained with the default *period* = 3 while the non-coding model was constrained to *period* = 1. In the testing step, the coding and non-coding ICMs were used to score the sequences using the glimmer3 program with the *linear* and *multifasta* options. The ICM metric score was computed as the log-ratio of the coding and non-coding likelihoods.

#### Z curve

The Z curve score for a sequence of DNA is a linear combination of 189 frame-specific mono-, di-, and tri-nucleotide occurrence frequencies [2],[72]. The weights assigned to these frequencies are trained by Fisher linear discriminant analysis on the frequency vectors computed from the coding and non-coding sequences in the training set, which we carried out using MATLAB with default settings.

### Hybrid Metrics

We created hybrid metrics by combining the pre-computed scores of the input metrics using linear discriminant analysis (LDA) and a support vector machine (SVM). In both cases, prior to combination, the scores of each input metric were normalized to have zero mean and unit variance across the entire dataset. The normalized scores from each input metric were then used as feature vectors representing each sequence in the dataset.

We trained and applied the hybrid metrics using four-fold cross-validation. We applied LDA with default settings in MATLAB. For SVM, we used SVM*^light^* 4.00 [73] with a linear kernel and default cost parameters. We used the prediction confidence computed by the svm_classify program as the SVM hybrid metric score for each sequence.

### Multidimensional Scaling

Multidimensional scaling (MDS) takes a high-dimensional matrix of pairwise similarities between items (in our case, metrics), and assigns each item to a point in a low-dimensional space (in our case, two dimensions for visualization), such that the distance between any two points approximately represents the dissimilarity of the corresponding items. We applied MDS to generate the visualization in Figure 2A using the R function cmdscale with default parameters. We defined the similarity between two metrics as *S*(*i*, *j*) = cor(*R_i_*, *R_j_*), where *R_i_* is the vector of ranks of the known exons according to the scores computed by metric *i*. For example, if the known exons are ordered in some way *E*_1_, *E*_2_, *E*_3_, and metric *i* assigns them scores *M_i_*([*E*_1_, *E*_2_, *E*_3_]) = [0.2,1.0,−0.5], then *R_i_* = [3],[1],[2].

## Supporting Information

Figure S1 Comparison of pairwise comparative metrics with *D. ananassae* as the informant species. Pairwise comparisons using the metrics we studied did not in general outperform the best single sequence metric (Z curve), although CSF and KA/KS achieve higher specificity.
(0.17 MB PDF)

Figure S2 Comparison of alignment depth provided by MULTIZ and Mercator/MAVID alignments. Shown on each plot is the cumulative proportion of regions in our dataset that have a certain number of species aligned (top) and the total branch length of those species (bottom), in the MULTIZ (red) or Mercator/MAVID (blue) alignments. For each region, an informant species was considered to align if at least 50% of the *D. melanogaster* nucleotides were aligned to an informant nucleotide (as opposed to gaps). The total branch lengths for the species aligning to each region were computed by taking the corresponding subtree of the neutral tree shown in Figure 3. In all cases, the MULTIZ alignments tend to align more species than the Mercator/MAVID alignments, consistent with their somewhat higher overall sensitivity (see Figure S3). (These results were generated from static genome alignment sets, and may not be representative of what is possible with the two approaches under different parameter settings.)
(0.23 MB PDF)

Figure S3 Comparison of discovery power provided by MULTIZ and Mercator/MAVID alignments. (Top) The MULTIZ alignments lead to higher sensitivity than the Mercator/MAVID alignments. The Mercator/MAVID alignments can lead to slightly higher specificity, but only at low sensitivities (<60%). (Bottom) The two alignments overall lead to concordant sets of detected exons, with >93% of exons detected in either alignment detected in both alignments. Although the MULTIZ alignments have higher overall sensitivity, the Mercator/MAVID alignments do uniquely allow the detection of ∼1.5% of exons. (These results were generated from static genome alignment sets, and may not be representative of what is possible with the two approaches under different parameter settings.)
(0.31 MB PDF)

Figure S4 Pairwise discovery power for RFC with different informants. More closely related species tend to yield higher sensitivity, while more distant species yield higher specificity.
(0.15 MB PDF)

Table S1 Gene detection rates within Gene Ontology (GO) categories. Each entry shows the percentage of genes with at least one exon detected at a fixed exon sensitivity cutoff for each metric.
(0.08 MB XLS)

Text S1 Information about access to test dataset coordinates, sequences, and alignments, and metric score data.
(0.03 MB DOC)
