# Supplementary figures and images for: Performance and Scalability of Discriminative Metrics for Comparative Gene Identification in 12 Drosophila Genomes
Source: PLoS Comput Biol. 2008 Apr 18;4(4):e1000067. doi: 10.1371/journal.pcbi.1000067 (PMC2291194; doi:10.1371/journal.pcbi.1000067)

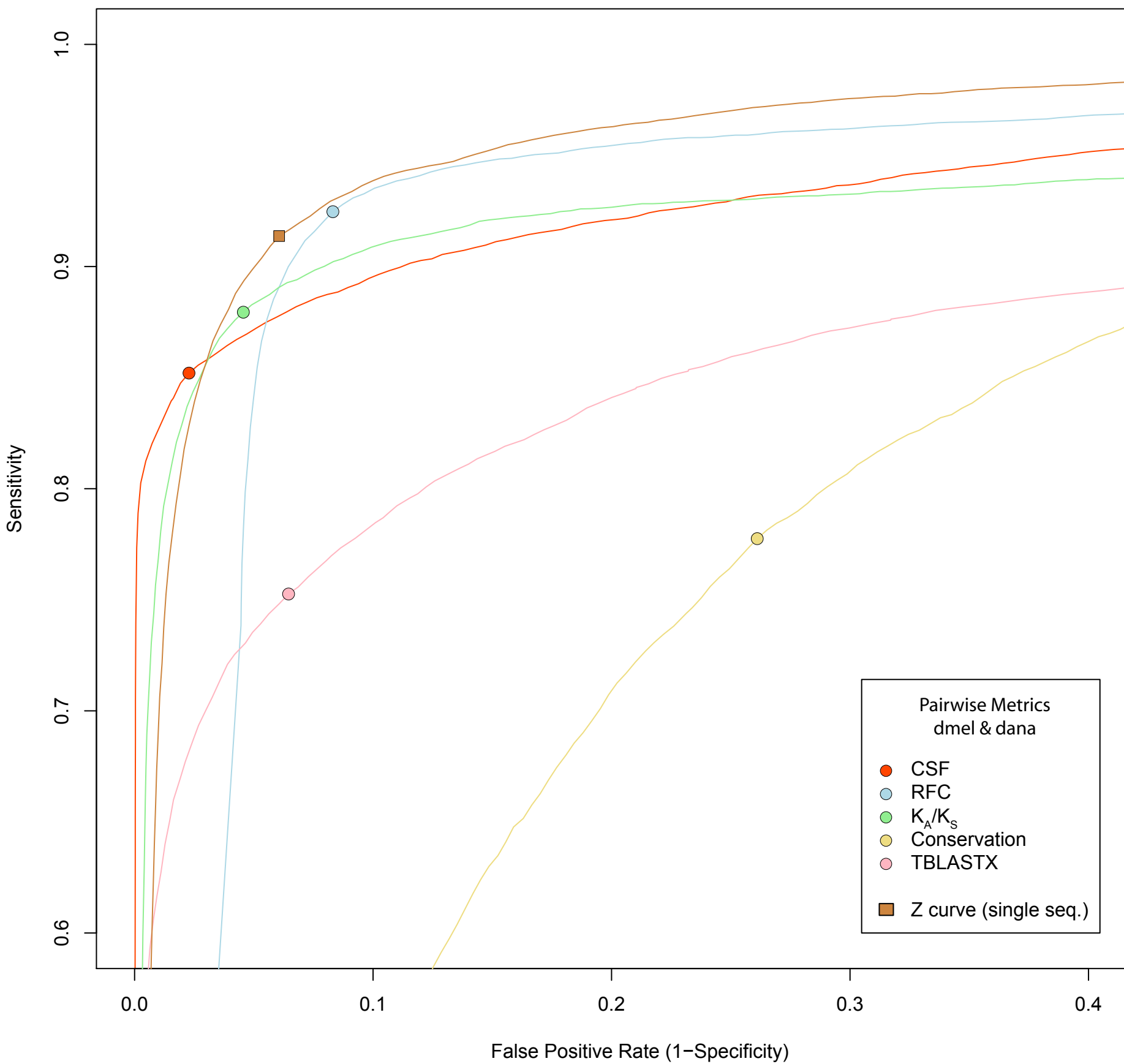

Supplement: Figure S1 — Comparison of pairwise comparative metrics with D. ananassae as the informant species. Pairwise comparisons using the metrics we studied did not in general outperform the best single sequence metric (Z curve), although CSF and KA/KS achieve higher specificity. (0.17 MB PDF) [file pcbi.1000067.s001.pdf]

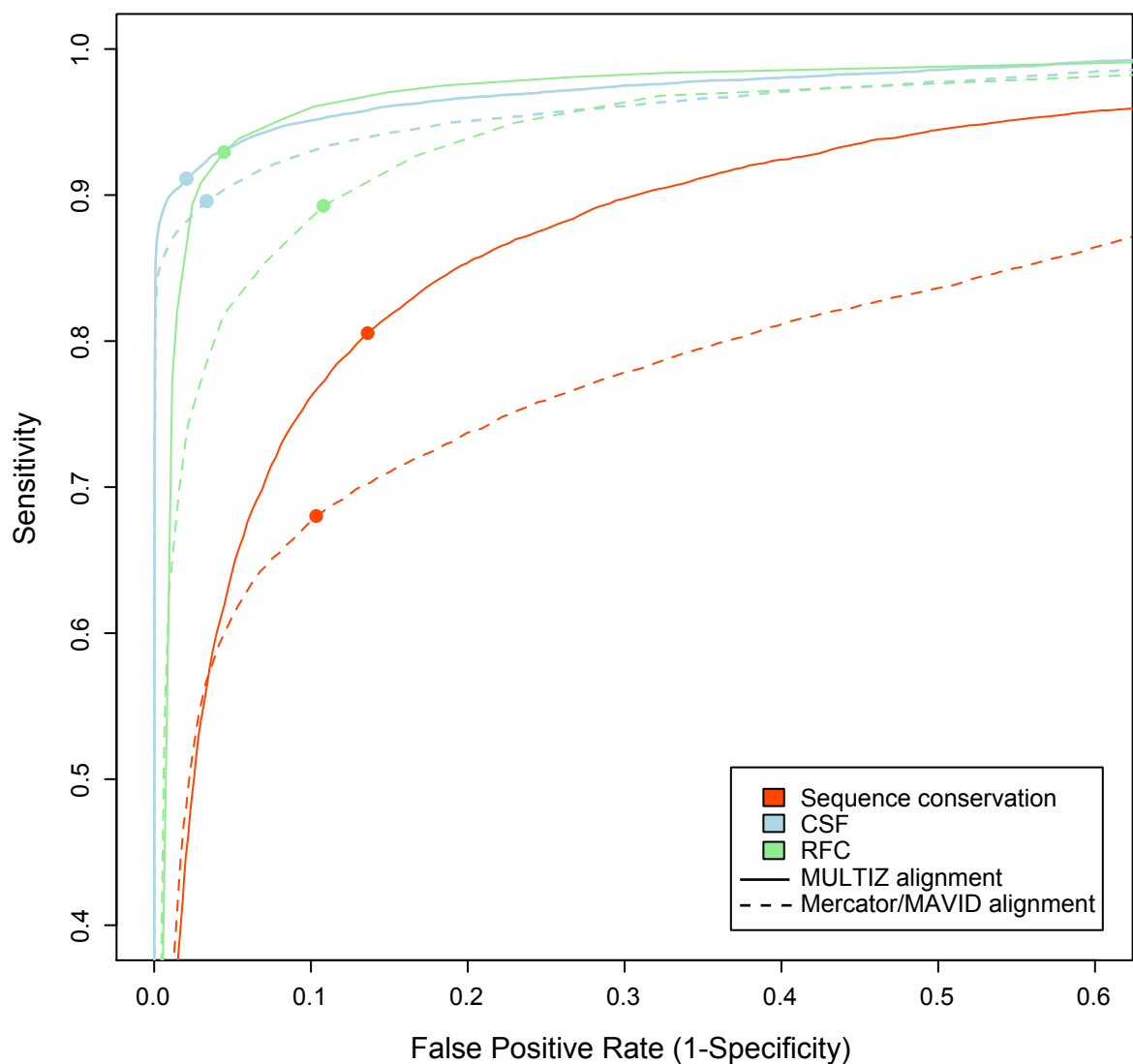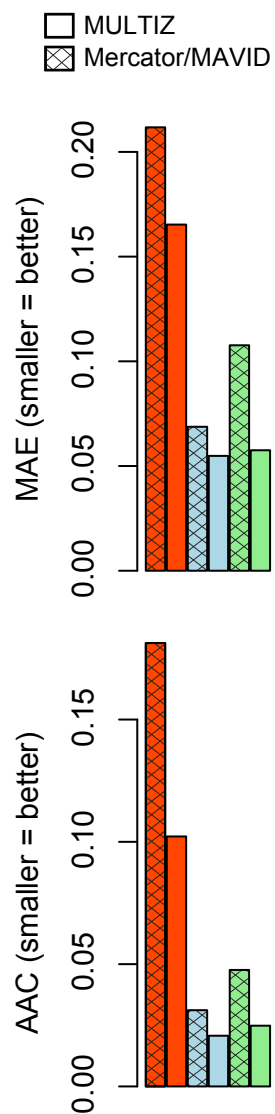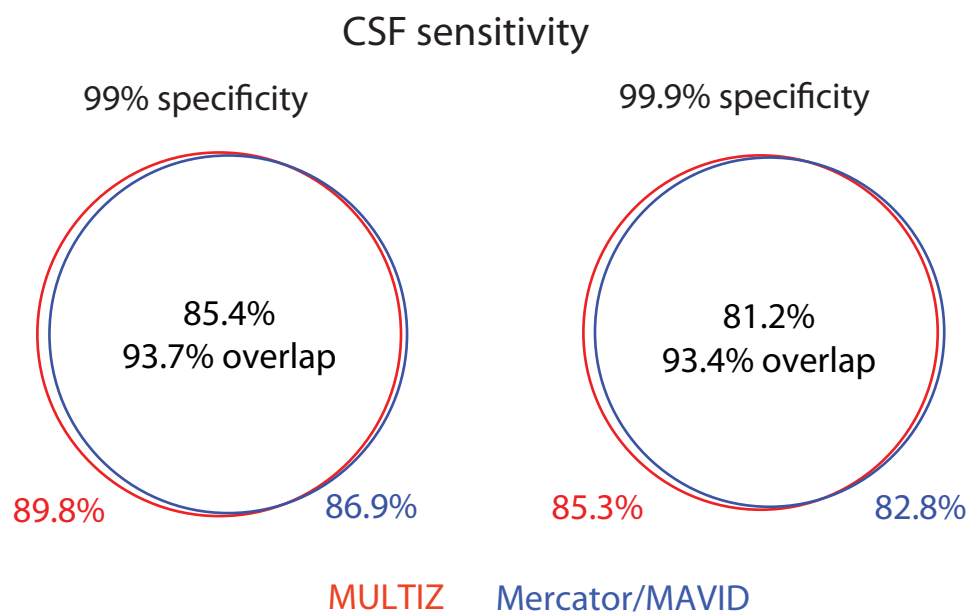

Supplement: Figure S3 — Comparison of discovery power provided by MULTIZ and Mercator/MAVID alignments. (Top) The MULTIZ alignments lead to higher sensitivity than the Mercator/MAVID alignments. The Mercator/MAVID alignments can lead to slightly higher specificity, but only at low sensitivities (<60%). (Bottom) The two alignments overall lead to concordant sets of detected exons, with >93% of exons detected in either alignment detected in both alignments. Although the MULTIZ alignments have higher overall sensitivity, the Mercator/MAVID alignments do uniquely allow the detection of ∼1.5% of exons. (These results were generated from static genome alignment sets, and may not be representative of what is possible with the two approaches under different parameter settings.) (0.31 MB PDF) [file pcbi.1000067.s003.pdf]

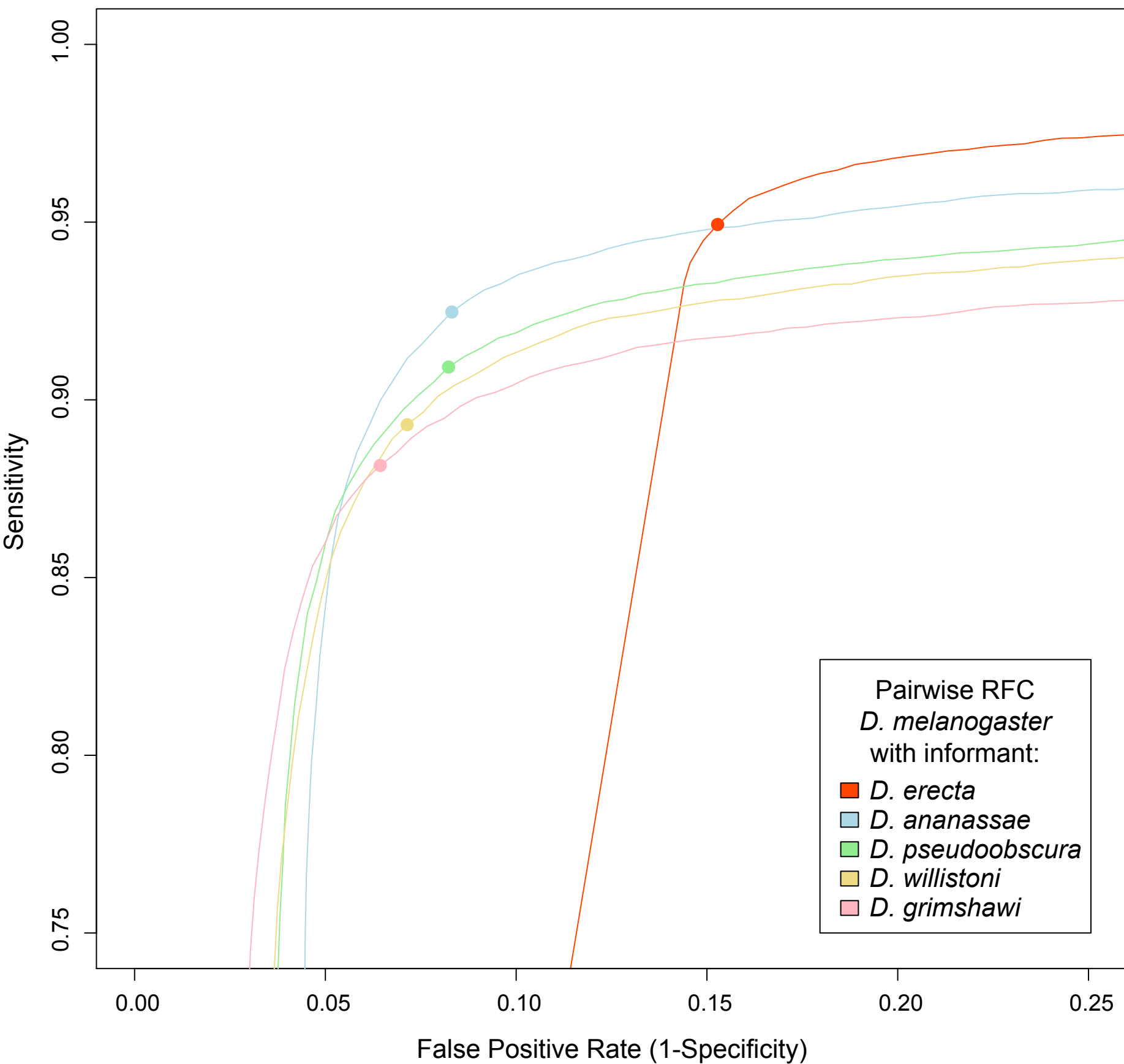

Supplement: Figure S4 — Pairwise discovery power for RFC with different informants. More closely related species tend to yield higher sensitivity, while more distant species yield higher specificity. (0.15 MB PDF) [file pcbi.1000067.s004.pdf]
