# Supplementary material for: Performance and Scalability of Discriminative Metrics for Comparative Gene Identification in 12 Drosophila Genomes
Source: PLoS Comput Biol. 2008 Apr 18;4(4):e1000067. doi: 10.1371/journal.pcbi.1000067 (PMC2291194; doi:10.1371/journal.pcbi.1000067)
Supplement: Figure S2 — Comparison of alignment depth provided by MULTIZ and Mercator/MAVID alignments. Shown on each plot is the cumulative proportion of regions in our dataset that have a certain number of species aligned (top) and the total branch length of those species (bottom), in the MULTIZ (red) or Mercator/MAVID (blue) alignments. For each region, an informant species was considered to align if at least 50% of the D. melanogaster nucleotides were aligned to an informant nucleotide (as opposed to gaps). The total branch lengths for the species aligning to each region were computed by taking the corresponding subtree of the neutral tree shown in Figure 3. In all cases, the MULTIZ alignments tend to align more species than the Mercator/MAVID alignments, consistent with their somewhat higher overall sensitivity (see Figure S3). (These results were generated from static genome alignment sets, and may not be representative of what is possible with the two approaches under different parameter settings.) (0.23 MB PDF) [file pcbi.1000067.s002.pdf]

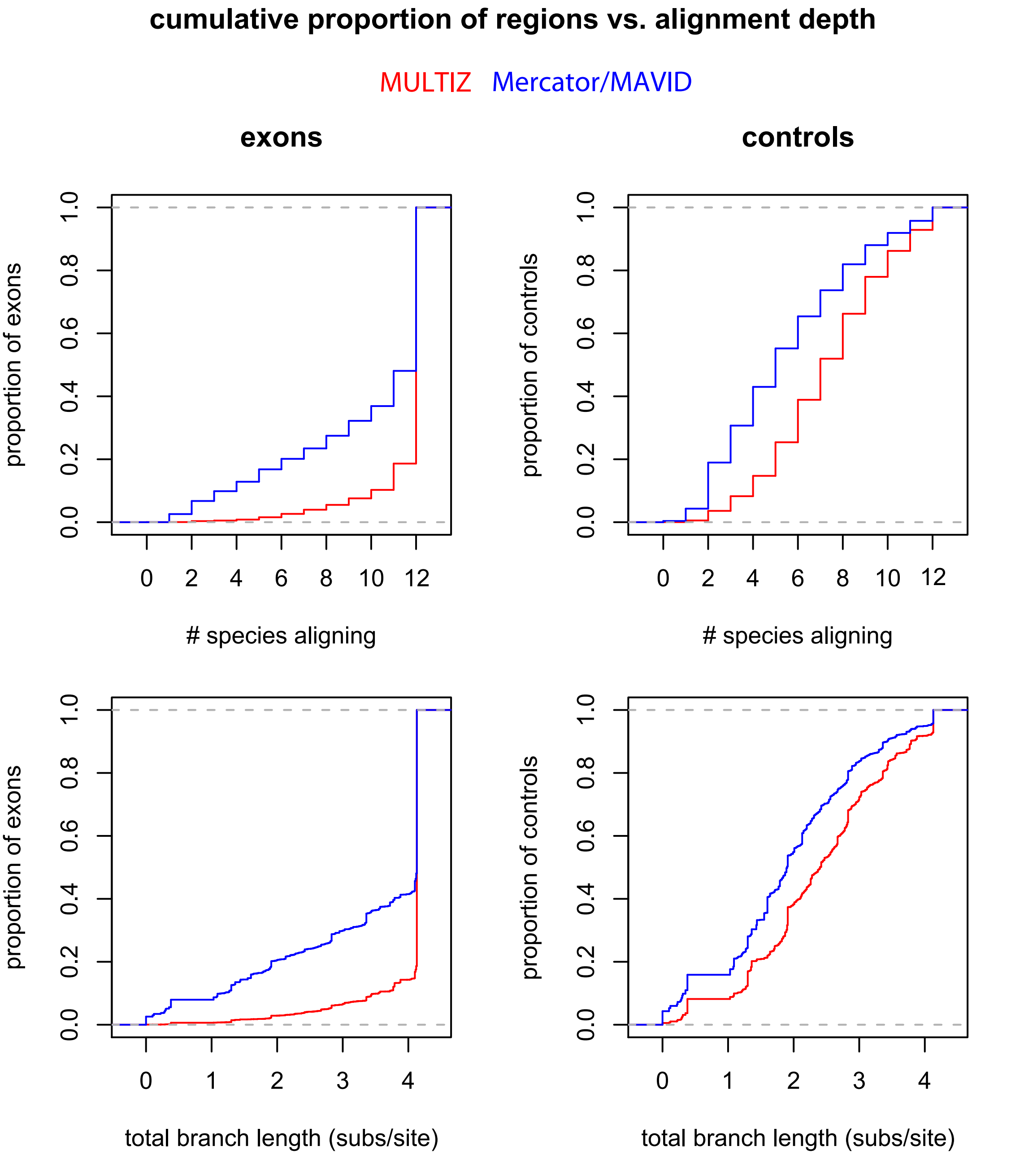
cumulative proportion of regions vs. alignment depth
MULTIZ
Mercator/MAVID
exons
controls
proportion of exons
proportion of controls
0.0
0.2
0.4
0.6
0.8
1.0
0
2
4
6
8
10
12
species aligning
0
1
2
3
4
total branch length (subs/site)
