# Supplementary material for: Performance and Scalability of Discriminative Metrics for Comparative Gene Identification in 12 Drosophila Genomes
Source: PLoS Comput Biol. 2008 Apr 18;4(4):e1000067. doi: 10.1371/journal.pcbi.1000067 (PMC2291194; doi:10.1371/journal.pcbi.1000067)
Supplement: Text S1 — Information about access to test dataset coordinates, sequences, and alignments, and metric score data. (0.03 MB DOC) [file pcbi.1000067.s006.doc]

Supporting Text S1 for Lin, Deoras, Rasmussen and Kellis (2008)
Performance and scalability of discriminative metrics for comparative gene identification in 12 Drosophila genomes

Additional supplemental data can be found on our web site,

<http://compbio.mit.edu/fly/metrics/>

- Test dataset
  - Identifiers and genomic coordinates
  - Sequences
  - Alignments
- Metric scores on the dataset
  - Scores for 12-genome comparative and single-sequence metrics
  - Scores for pairwise comparative metrics with different informants
  - Scores for multi-species comparative metrics with different informant subsets
